# Thermal Modulation of Musalais Wine Characteristics: Volatile Profiles and Chemical Composition at Different Brix Levels

**DOI:** 10.3390/foods14172956

**Published:** 2025-08-25

**Authors:** Buhailiqiemu Abudureheman, Minqiang Guo, Jianlin Zhang, Lin Chen, Qian Li, Tiantian Long, Zhuanzhuan Lv, Junli Huang, Dandan Fang, Luxi Jiang, Xingqian Ye, Haibo Pan

**Affiliations:** 1 Key Laboratory for Quality Testing of Musalais, Center for Experimental Instruction in Food Safety and Nutrition, Xinjiang Institute of Technology, Aksu 843000, China; buhalqam.a@163.com (B.A.); gmqyx123@163.com (M.G.); zjl950702@163.com (J.Z.); nei3677585@163.com (L.C.); 18599340828@163.com (Q.L.); ltt1016@icloud.com (T.L.); 18409496308@163.com (Z.L.); mengduud@163.com (J.H.); fdc2015087202112@126.com (D.F.); luxijiang123@yeah.net (L.J.); 2 College of Biosystems Engineering and Food Science, National-Local Joint Engineering Laboratory of Intelligent Food Technology and Equipment, Zhejiang Key Laboratory for Agro-Food Processing, Zhejiang Engineering Laboratory of Food Technology and Equipment, Fuli Institute of Food Science, Zhejiang University, Hangzhou 310058, China; 3 Innovation Center of Yangtze River Delta, Zhejiang University, Jiaxing 314102, China; apanhaibo@126.com

**Keywords:** Musalais wine, flavor component, fermentation methods, physicochemical

## Abstract

This study investigated the effects of fermentation temperatures (22 °C, 25 °C, 28 °C) and concentrations of grape juice Brix (26 °, 29 °, 32 °) on the physicochemical and aroma profiles of Musalais wine, a traditional fermented alcoholic beverage from Xinjiang, China. The results indicated that higher fermentation temperatures (28 °C) increased total acidity (TA) and residual sugar content (RSC), whereas lower temperatures (22 °C) resulted in higher pH, phenolic content, and anthocyanin content. Ethanol content reached its peak at 25 °C, particularly in Musalais wines produced from 29 Brix of concentrated grape juice. GC-IMS analysis identified 50 volatile organic compounds (VOCs), with esters (30%), alcohols (22%), and ketones (12%) dominating the aroma profile. Wines fermented at 22 °C exhibited the most complex VOC profiles, characterized by fruity esters (ethyl propanoate) and caramel-like ketones (4-methyl-2-pentanone). In contrast, fermentation at 28 °C produced simpler alcohol-dominated aroma profiles. Multivariate analysis (PCA and PLS-DA) confirmed distinct clustering based on temperature, with 19 key markers (ethyl 2-methylpentanoate, 3-octanone) differentiating the Musalais wines. Correlation analysis revealed strong relationships between ethanol, TA, RSC, and specific VOCs. Hierarchical clustering grouped the wines into two categories: those fermented at 22 °C (fruity and rich in complexity) and those fermented at 25–28 °C (alcoholic and simpler profiles). These findings demonstrate that fermentation temperature significantly impacts Musalais wine quality, with 22 °C being optimal for aroma complexity, while 25 °C provided a balance between ethanol production and antioxidant retention. Brix levels of concentrated grape juice modulated acidity and sweetness. This study offers practical insights for optimizing Musalais wine production through controlled fermentation conditions.

## 1. Introduction

Musalais wine, a traditional alcoholic fermented beverage, is crafted from the juice of native grape varieties like Hetian hong and Awatihong and is predominantly found in the southern part of Xinjiang, China. The creation of Musalais involves a meticulous process that includes washing grapes, juice extraction, concentration via boiling, and natural fermentation. Musalais wine is usually fermented at room temperature at 20–28 °C, which takes more than two weeks. Commercially manufactured Musalais wine is usually fermented for a short period (5 to 7 d) at 26 °C using yeast.

Volatile compounds are abundant in grape skins. The contact between pulp and skin during juice extraction results in higher concentrations of these compounds [1]. Volatile compounds determine the quality of wine because of their influence on the aroma sensory profile. Spranger et al. (2004) [2] estimated that different grapes and wines contain around 1000 aroma compounds in different amounts. Tang et al. (2025) [3] reported that a total of 1364 compounds, classified into 20 distinct categories, were identified during the fermentation of Hotan red Musalais wine (prepared from raw grapes at 17 °Brix, concentrated to 28 °Brix, and naturally fermented at 25 °C for 15 d). The predominant metabolites included 1-amino-1-cyclobutanecarboxylic acid, 3-amino-4-hydroxy-N-methylbenzenesulfonamide (gramine), pyrogallol, phloroglucinol, malic acid, [(4-methylbenzyl)sulfanyl]acetic acid, erythronolactone, and flunixin. Microbes such as *Lactiplantibacillus*, *Rhodotorula*, *Thermoascus*, *Aspergillus*, *Leuconostoc*, *Mycobacterium*, *Blautia*, and *Hungatella* are key contributors to flavor and nutrient formation. Different aromas contribute distinctively to the overall flavor profile of wine. Therefore, analyzing grape varieties and quantifying volatile compounds generated during fermentation can aid in producing Musalais wine with superior aroma and taste. Wine quality is further influenced by fermentation temperature and microbial terroir, which encompasses factors such as grape cultivation, brewing environment, grape characteristics, and cellar conditions. Higher fermentation temperatures (25–30 °C) are typically employed to enhance phenolic compound extraction, resulting in wines with deeper color and more robust structure [4,5]. In contrast, fermentation at lower temperatures (below 15 °C) proceeds more slowly but improves sensory quality. Consequently, fermentation temperature plays a critical role in modulating volatile compound composition.

There are studies on the influence of the fermentation temperature on the combination of volatile compounds in wines [1,3,6,7,8,9,10,11]. However, there are no reports about the effects of the Brix level of grape juice after concentrating at high temperature and fermentation temperature on aroma-active compounds in Musalais wine. This study examines how the fermentation temperature (22–28 °C) and Brix level (26–32 °) of grape juice affect Musalais wine’s physicochemical and aroma properties. Using GC-IMS and multivariate analysis, we identified key VOCs and established optimal conditions balancing aroma, ethanol, and antioxidants.

## 2. Materials and Methods

### 2.1. Fermentation Assays

Hetian hong grapes were collected manually in the Hetian region of Xinjiang (Southwest Xinjiang) in October 2024 at their optimum ripening degree: 21 °Brix. After washing, de-stemming, and crushing, the grape juice was concentrated at a high temperature (100 °C) to values of Brix that reached 26 °, 29 °, and 32 °, separately, using a stainless-steel boiling pot.

After cooling to room temperature, the concentrated grape juice was naturally fermented at three different temperatures (22 °C, 25 °C, and 28 °C) in 50 L fermentation tanks at the Key Laboratory for Quality Testing of Musalais, College of Food Science and Engineering, Xinjiang Institute of Technology.

All assays were conducted in stainless steel fermentation tanks across the tested Brix level of concentrated grape juice and fermentation temperatures. Temperature control was achieved using a water-cooled jacket system that partially covered the tanks. Upon completion of fermentation, the Musalais wines were filtered, bottled, pasteurized at 80 °C for 30 min, and stored at 20 °C.

### 2.2. Determination of the Basic Physicochemical Indicators of Musalais Wine

#### 2.2.1. Determination of Total Acidity (TA)

TA was determined using the potentiometric method [12], titrating a sample with 0.1 M NaOH solution to obtain pH 7.

#### 2.2.2. Alcohol Concentration

The alcohol concentration in Musalais wine was measured following the method of Cai et al. (2021) [13]. After fermentation, samples were distilled, and the resulting distillate was diluted to 100 mL with distilled water. The density was then determined, and the ethanol concentration was calculated using reference tables. To measure the real extract content, the distillation residues were transferred quantitatively to a 100 mL volumetric flask, diluted to the same volume with distilled water, and the procedure was repeated.

#### 2.2.3. pH

The pH was determined in accordance with the AOAC guidelines [14]. After calibrating the digital scale pH meter (ATC, Shenzhen, China), the electrodes were inserted into 20 mL of the Musalais wine and the results were recorded in triplicate.

#### 2.2.4. Residual Sugar Content

Reduced sugars were measured following the method of Yadav et al. (2022) [15]. Briefly, Fehling solutions A and B were mixed and heated, then methylene blue was added. The sample solution was titrated against Fehling solution until a brick-red endpoint appeared, indicating the disappearance of the blue color. The volume of the sugar solution used was recorded and was used to calculate the residual sugar percentage using the following equation:

(1) $$\mathrm{Residual Sugar}\;(\%)=\frac{\mathrm{Fehling factor}\times \mathrm{dilution}\;(mL)}{\mathrm{Volume of sample}\;(mL)\times \mathrm{titre}\;(mL)}\times 100$$

#### 2.2.5. Vitamin C (VC)

VC was identified and quantified following the method of Varo Et Al. (2022) [16]. Briefly, 0.7 mL of 4.5% metaphosphoric acid was added to 0.7 mL of Musalais wine. The mixture was sonicated for 5 min, after which 1 mL of the supernatant was mixed with 0.2 mL of DTT solution. The sample was then kept in the dark for 2 h to allow the complete conversion of dehydroascorbic acid to L-ascorbic acid. Following conversion, the sample was filtered through a 0.45 μm nylon membrane. Quantification was performed using a Waters e2695 HPLC system (Waters Technologies Co., Ltd., Shanghai, China) equipped with a GL Inertsil ODS-3 column (250 × 4.6 mm, 5 μm; Anpel Laboratory Technologies, Shanghai, China). The mobile phase consisted of (A) 0.2 M KH_2_PO_4_ (pH 2.3–2.4) and (B) acetonitrile, with isocratic elution at a flow rate of 1.0 mL/min. Detection was performed at 243 nm with an injection volume of 20 μL. 

### 2.3. Analysis of Polyphenol Profile

#### 2.3.1. Total Polyphenol Content (TPC)

The TPC of the Musalais wine was quantified using the Folin–Ciocalteu assay [1]. A calibration curve with R^2^ = 0.9977 was constructed using gallic acid solutions (0.02–0.1 mM). The TPC data were reported as mM gallic acid equivalents (GE/L).

#### 2.3.2. Total Flavonoid Content (TFC)

TFC of the Musalais wine was determined using aluminum chloride complex forming assay [17]. The calibration curve for rutin (0.02–0.1 mg/L) had R^2^ = 0.9938.

#### 2.3.3. Total Anthocyanin Content

The total anthocyanin content was determined using the pH differential method [18]. Prior to analysis, Musalais wine samples were centrifuged or filtered to remove particulate matter. Equal volumes of each sample were separately mixed with pH 1.0 and pH 4.5 buffer solutions, then equilibrated in the dark for 30 min to ensure complete structural conversion of anthocyanins. Absorbance measurements were taken at 520 nm (anthocyanin peak) and 700 nm (turbidity correction), using the respective buffers as blanks. The corrected absorbance (A) was calculated using Equation (2):

(2) $$A=(A_{520}-A_{700}){pH}_{1.0}-(A_{520}-A_{700}){pH}_{4.5}$$

Total anthocyanin content was expressed as cyanidin-3-glucoside equivalents (mg/L) using Formula (3):

(3) $$\mathrm{Anthocyanin}\;(mg/L)=\frac{A\times MV\times DF\times 1000}{\varepsilon \times l}$$

where MW is the molecular weight of cyanidin-3-glucoside (449.2 g/moL); DF is the dilution factor; ε is the molar extinction coefficient (26,900 L·mol^−1^·cm^−1^); and l is the path length (1 cm)

### 2.4. Analysis of Volatile Compounds by GC-IMS

GC-IMS analysis was performed using a GC-IMS (GC-IMS, FlavourSpec^®^, G.A.S, Dortmund, Germany) and an MXT-WAX capillary column (30 m × 0.53 mm × 1 μm) (Restek, Bellefonte, PA, USA), following the experimental method of Jiang et al. (2024) [19] with slight modifications. Each Musalais wine sample was prepared by adding 0.5 mL into a 20 mL headspace vial, sealed with a magnetic cap, and incubated at 60 °C for 25 min. Subsequently, 200 mL of the headspace was injected into the sampler using a syringe at a flow rate of 60 mL/min at 85 °C. The chromatographic column temperature was set at 60 °C, while the drift tube was adjusted to conditions of 45 °C. The flow rate of the drift gas was set at 150 mL/min. High-purity nitrogen gas (purity of 99.99%) was used as the carrier gas, with the flow rate of the gas chromatographic column set to the following: 2 mL/min for 2 min, 10 mL/min for 10 min, 50 mL/min for 15 min, 100 mL/min for 20 min, and 150 mL/min for 30 min. The retention index (RI) of VOCs was calculated using n-ketones C_4_–C_9_ as a reference. VOCs were identified by comparing the ion drift time and retention time with standards in the GC-IMS library. Each sample was analyzed once, and each VOC was relatively quantified based on peak area. The n-ketone C_4_–C_9_ standard solution was purchased from Sinopharm Chemical Reagent Co., Ltd., Shanghai, China.

### 2.5. Statistical Analysis

The antioxidant activities of the Musalais wine were reported as means ± standard error (SE) and tested for statistical difference at a 95% confidence by analysis of variance in a general linear model (GLM) followed by Tukey’s test (SPSS Statistics 18.0). The one-way analysis of variance (ANOVA) was performed using Tukey’s method with a 95% confidence level to determine significant differences in the comparison of results. A qualitative analysis of volatile compounds in the samples was conducted using the GC × IMS Library Search NIST database and IMS database built into VOCal software. The Reporter plugin was used to view the sample two-dimensional spectra and analyze the differences between spectra; SIMCA 14.1 software was used for principal component analysis (PCA) and partial least squares discriminant analysis; Pearson’s correlation analysis was used to determine the correlation, which was carried out through the website at https://www.chiplot.online/, accessed on 8 May 2025. The Gallery Plot plugin was used to draw fingerprint plots; Origin 2022 was used for bar charts and pie charts.

## 3. Results and Discussion

### 3.1. Basic Physicochemical Properties of Musalais Wine

**Figure 1:**
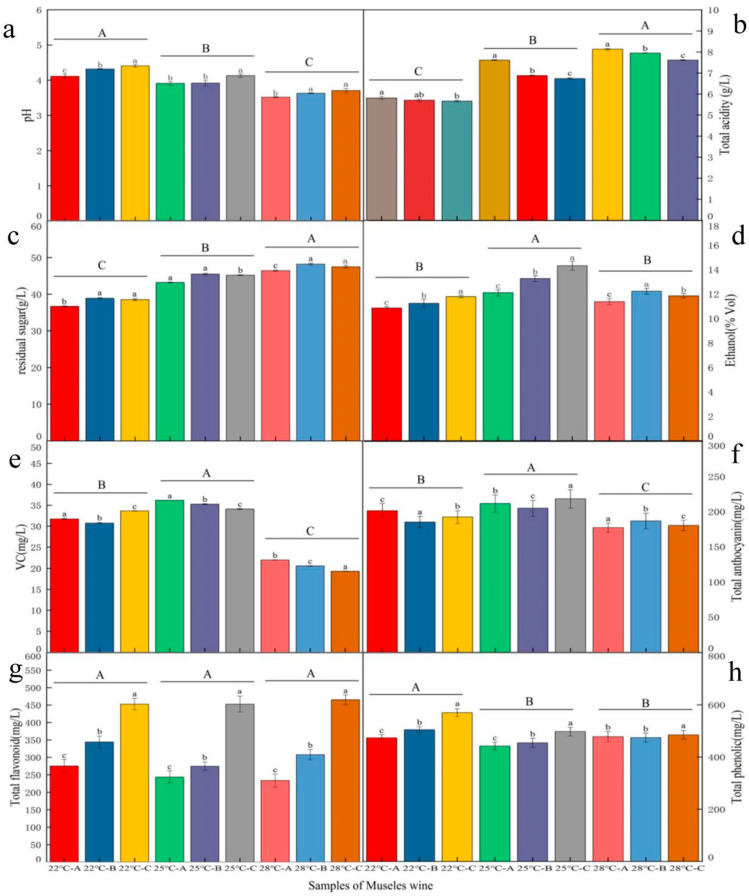
Chemical and antioxidant properties of Musalais wine produced by different fermentation temperatures after concentrating the grape juice to different Brix levels. Note: Fermentation temperatures of 22 °C, 25 °C, and 28 °C were used to concentrate grape juice at 100 °C to different Brix levels of 26 ° (A), 29 ° (B), and 32 ° (C). (**a**): pH; (**b**): Total acidity (g/L); (**c**): residul sugar (g/L); (**d**): Ethanol (% Vol); (**e**): VC (mg/L); (**f**): Total anthocyanin (mg/L); (**g**): Total flavonoid (mg/L); (**h**): Total phenolic (mg/L). Different lowercase letters denote statistically significant differences between the Brix at the same fermentation temperature of the total acidity, residual sugar content, pH, ethanol, VC, total anthocyanin, total phenolic content, and total flavonoid content. Different capital letters denote statistically significant differences among the fermentation temperatures of the total acidity, residual sugar content, pH, ethanol, VC, total anthocyanin, total phenolic content, and total flavonoid content. All statistics were determined by Duncan’s multiple range test.

Significant differences (*p* < 0.05) were observed in pH, total acidity (TA), residual sugar content (RSC), ethanol content, vitamin C (VC) levels, total anthocyanins, flavonoids, and total phenolics among the Musalais wines fermented at different temperatures (22 °C, 25 °C, 28 °C) and with varying Brix levels of concentrated grape juice (26 °, 29 °, 32 °) (hereafter referred to as Brix juice or Brix) (Figure 1).

Total acidity (TA) and residual sugar content (RSC) exhibited positive correlations with fermentation temperature (Figure 1b,c). Significantly higher values (*p* < 0.05) were observed at 28 °C compared to 25 °C and 22 °C, in the following order: 28 °C > 25 °C > 22 °C. This temperature-dependent increase can be attributed to enhanced hydrogen ion dissociation at elevated temperatures, leading to greater acidity [20].

The Brix level also significantly affected TA and RSC. TA demonstrated an inverse relationship with Brix level, reaching its maximum value (8.24 ± 0.20 g/L) at 28 °C when using 26 °Brix juice. In contrast, RSC showed the opposite trend, with the highest levels obtained using 29 °Brix juice (29 ° > 32 ° > 26 °; Figure 1b).

In contrast, pH exhibited an inverse relationship with TA (Figure 1a), showing significantly higher values at lower fermentation temperatures (22 °C > 25 °C > 28 °C) (*p* < 0.05). Furthermore, pH demonstrated a positive correlation with Brix levels, consistently reaching its maximum values with 32 °Brix juice across all tested temperatures (22 °C, 25 °C, and 28 °C).

Ethanol content (% *v*/*v*) showed significant variation depending on both fermentation temperature and Brix level. Maximum ethanol production was achieved at 25 °C of fermentation temperature, with progressively lower yields at 28 °C and 22 °C (25 °C > 28 °C > 22 °C), aligning with findings by Wu et al. (2024) [7] and Li et al. (2025) [8]. This research showed a distinct Brix-dependent pattern emerging: while ethanol content generally increased with the Brix level between 22 ° and 25 °, optimal production at 28 °C occurred specifically at 29 °Brix. The ethanol content of Musalais wine of the 29 °Brix juice fermented at 28 °C significantly exceeded (*p* < 0.05) that of both the lower (26 °) and higher (32 °) Brix levels.

Phenolic compounds, particularly anthocyanins, are crucial determinants of wine quality, significantly influencing both its sensory attributes and bioactive properties. Although fermentation temperature showed no significant effect on flavonoid content (*p* > 0.05) (Figure 1g)—a finding consistent with the report by Du et al. (2024) [21]—the juice Brix significantly affected flavonoid levels (*p* < 0.05), with the highest concentrations observed at 32 °Brix.

Total phenolic content (TPC), total anthocyanins (TAC), and vitamin C (VC) were significantly higher in the Musalais wine at 22 °C and 25 °C compared to 28 °C (*p* < 0.05) (Figure 1e,f,h). The fermentation condition, grape variety, processing methods, and microbial activity affect the TPC and TAC of wine [22,23]. Ampofo et al. (2020) showed that of the 25 °C, 30 °C, 35 °C, and 40 °C temperatures, *Phaseolus vulgaris* sprouts showed the highest total phenolic acid, total flavonoid, and anthocyanin contents at 30 °C [24]. The reason may be that phenolic compounds inherently exhibit poor stability. Phenolic stability is temperature-dependent, as elevated temperatures accelerate molecular degradation [25], explaining the reduced levels of phenolics at higher fermentation temperatures. Notably, TPC increased with Brix, peaking at 32 °Brix; this is contrary to findings by Wu et al. (2024) [7], likely due to differences in grape varieties or fermentation protocols. Tang et al. (2025) [3] reported that microbial genera, including *Blautia*, *Leuconostoc*, *Lactiplantibacillus*, *Rhodotorula*, and *Thermoascus*, produce both greater quantities and varieties of metabolites, and genera such as *Thermoascus* drive this synergistic synthesis process in metabolites of glycine, acetamide, erythronolactone, glucuronic acid, fertaric acid, 1-amino-1-cyclobutanecarboxylic acid, and gramine. This may be because the high Brix level and the open fermentation environment provide multiple carbon sources for fermentation. These results highlight the critical role of microbial activity, Brix level, and temperature in modulating phenolic profiles during Musalais production.

### 3.2. Volatile Compounds Identified by GC-IMS

**Figure 2:**
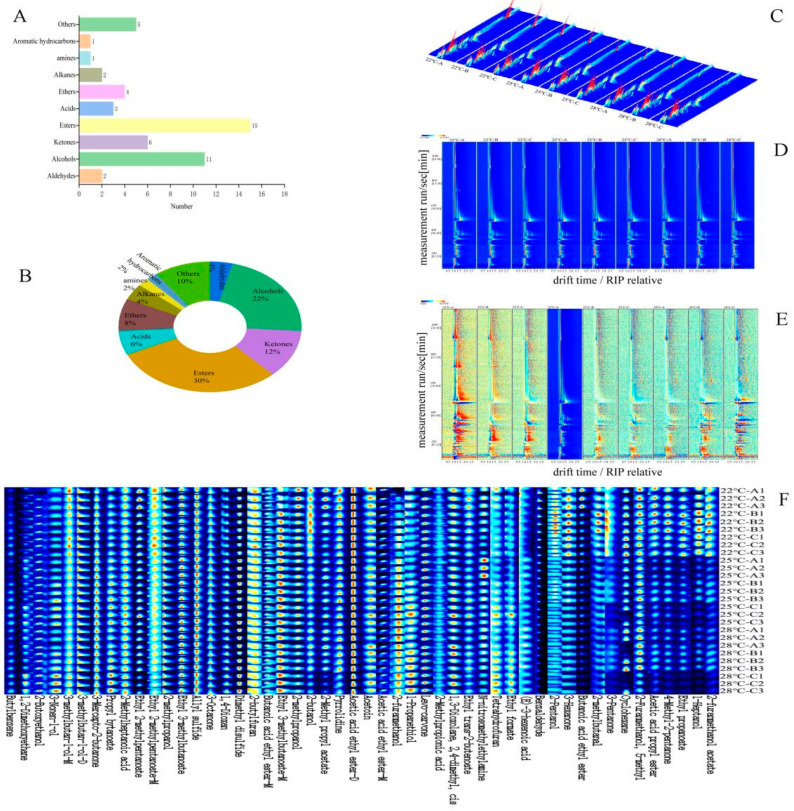
Characteristics of volatile flavor compounds in Musalais wine with different brewing processes, and the qualitative analysis of volatile compounds. (**A**) Number and percentage of volatile compounds; (**B**) proportion of volatile flavor compounds; (**C**) three-dimensional GC-IMS spectra; (**D**) two-dimensional GC-IMS spectra; (**E**) differential GC-IMS spectra and two-dimensional GC-IMS spectra; (**F**) fingerprint spectrum of volatile compounds identified in Musalais wine samples by GC-IMS. Note: The colors represent the concentration of the substance, with white indicating a lower concentration, red indicating a higher concentration, and darker colors indicating a greater concentration.

The volatile compounds were also analyzed by GC-IMS to reveal the differences in volatiles in Musalais wines with different brewing processes. The results of the qualitative analysis of volatile flavor compounds were shown in Figure 2A,B and Table 1. In this study, a total of fifty volatiles were found in nine Musalais wine samples. The number and proportion of the 50 common volatile compounds composed: alcohols (11, 22%), ketones (6, 12%), esters (15, 30%), acids (3, 6%), aldehydes (2, 4%), ethers (4, 8%), alkanes (2, 4%), aromatic hydrocarbons (1, 2%), amines (1, 2%), and others (5, 10%). In terms of the percentages of each type of compound in the volatiles, esters, alcohols, ketones, and ethers represent a relatively large proportion, accounting for 72.0% of the total amount of volatiles identified (Figure 2B), which is the same as the results of Zhang et al. (2017) [26]. Zhang et al. (2017) [26] studied the aroma components in three differently clarified (turbid, semi-clarified, clarified) Musalais wines and found eighteen types of common compounds. Tang et al. (2025) [3] also found 20 kinds of compounds, which were mainly alcohols, esters, aldehydes, and ketones. The grape juice concentration process can destroy the original aromatic components present in grapes and grape juice, but various aromatic compounds such as alcohols and esters are produced during the concentration, natural fermentation, and aging, providing Musalais with rich aromas, including caramel, alcoholic, and ester-like scents [9,27].

### 3.3. Graph Analysis

**Figure 3:**
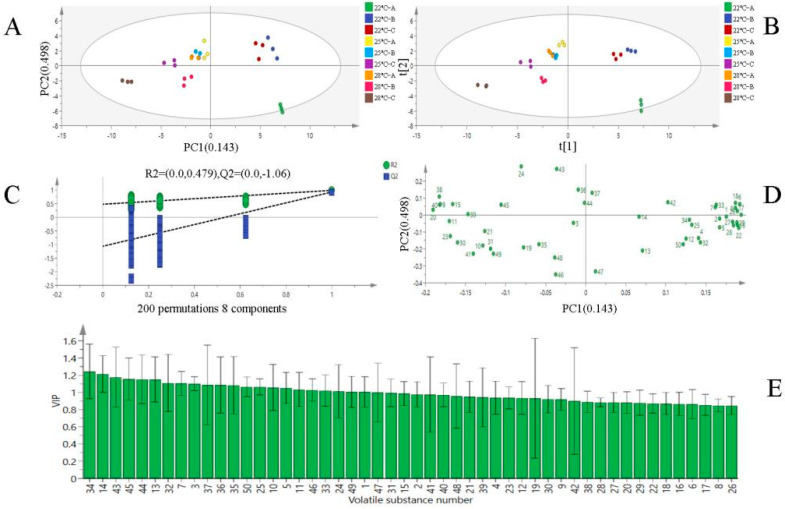
OPLS-DA analysis of Musalais wine after different fermentation treatments. PCA analysis (**A**,**B**); OPLS-DA score plot (**C**); confidence test results (**D**); VIP value distribution of volatile substances (**E**).

Utilizing a three-dimensional GC-IMS spectrum, differential analysis was performed on the VOCs of Musalais wine with different brewing processes, based on retention time, drift time, and peak intensity (Figure 2C). The three-dimensional spectrum of Figure 2C was projected onto a two-dimensional plane, and the top-down view is shown in Figure 2E. In Figure 2C, the X-axis represents retention time, the Y-axis represents drift time, and the Z-axis represents ion peaks. Each point in the spectrum represents a volatile organic compound. The shade of color indicates the concentration level, the blue represents the background, and the color represents the concentration of the substance, with white indicating a lower concentration and red indicating a higher concentration. Depending on the concentration and properties of the VOCs, the compound may produce one, two, or more spots (representing monomers, dimers, or trimers) [28]. Among the Musalais wine samples, 22 °C-B exhibits a relatively higher concentration of VOCs (Figure 2E). From Figure 3C,E, it can be clearly seen that there are significant differences in the GC-IMS characteristic spectra of VOCs between Musalais wine with different brewing processes.

To further compare the differences in VOCs among Musalais wine samples of varying sensory quality grades, fingerprint maps were generated from the signal peaks of each substance in the GC-IMS two-dimensional spectra to identify the characteristic peak regions of different liquor samples. The horizontal axis of the map represents the VOCs detected by GC-IMS, while the vertical axis represents different grades of Musalais wine samples. Each row displays all selected signal peaks for Musalais wine samples of that grade, and each column illustrates the concentration variations in a compound across different samples. The brightness of individual points indicates the content level of a specific volatile compound, with darker shades representing higher concentrations [29].

Figure 2F reveals significant differences in VOCs among Musalais wine samples produced at different fermentation conditions. All samples contained common VOCs, including 2-furanmethanol, 5-methyl-2-furanmethanol, ethyl acetate-D, acetoin, 1,3-dioxolane, 2,4-dimethyl-pyrrolidine, ethyl 3-methylbutenoate M, 2-butylfuran, allyl sulfide, ethyl 3-methylbutanoate, 2-methylpropanol, ethyl 2-methylpentanoate, 2-ethylheptanoic acid, 3-mercapto-2-butanone, and both D- and M-forms of 3-methylbutan-1-ol. The 22 °C-fermented Musalais wine showed the highest concentrations of VOCs, particularly esters (butanoic acid ethyl ester, acetic acid propyl ester, ethyl propanoate), ketones (3-hexanone, 4-methyl-2-pentanone, 3-pentanone), and alcohols (2-furanmethanol, 5-methyl-2-furanmethanol, 2-pentanol). The fruity, sweet, rum-like aromas of 4-methyl-2-pentanone and various ethyl esters, combined with caramel notes from furanmethanol derivatives, contributed to the distinctive fragrance of these Musalais wine samples [10]. Musalais wine samples fermented at 25 °C contained elevated levels of propanethiol (onion/garlic aroma), 2-furanmethanol (caramel), and ethyl formate (rum-like). The 28 °C-B, C and 25 °C-C samples showed increased concentrations of ethyl formate, tetrahydrofuran, propyl butanoate, and 3-nonen-1-ol, indicating the onset of oxidative changes as ethyl formate formation suggests acetaldehyde activity. These samples maintained some fruity characteristics but developed oxidative notes. The presence of propanethiol and 2-furanmethanol at both 28 °C and 25 °C created a balance between fruitiness and emerging sulfurous/solvent-like characteristics. The transition of the VOCs at 25 °C reflects a metabolic shift toward higher alcohol and sulfur compound production [30], with propanethiol indicating stress-related sulfur metabolism potentially due to nutrient limitations [31]. The Musalais wines fermented at 28 °C contained high levels of ethyl formate (sharp, rum-like), 3-nonen-1-ol (grassy), and propyl butanoate (subdued fruitiness), resulting in a profile dominated by oxidative, fusel, and herbal characteristics. This observation supports previous findings that temperatures exceeding 26 °C promote yeast autolysis and oxidative reactions while reducing ester formation and increasing aldehyde content [31]. It should be noted that compound concentration does not directly correlate with aroma intensity, as individual substances may exhibit different aromatic properties at varying concentrations [32,33,34]. The overall sensory quality depends on the harmonious balance of these aromatic components.

Alcoholic and fruity aromas of wine mainly come from alcohols, which are the second-largest group of aromatic compounds formed principally during natural fermentation after esters in fruit wines [35]. From Table 1, it can be seen that among the nine Musalais wine samples, the number of alcohols is relatively high and commonly shared alcohols include 2-methylpropanol, 2-pentanol, 2-butanol, 2-furanmethanol, 5-methyl, 1-heptanol, 2-furanmethanol, 1-propanethiol,3-nonen-1-ol, 3-methylbutan-1-ol-D, and 3-methylbutan-1-ol-M.

The esters in wine are mainly produced during the fermentation process, and they are responsible for fruity aromas [36]. In this research, esters are an important qualitative group, and a total of fifteen compounds have been identified, namely propyl butanoate, ethyl 3-methylbutanoate-D, butanoic acid ethyl ester-D, butanoic acid ethyl ester-M, ethyl 3-methylbutanoate-M, 2-methyl propyl acetate, 2-furanmethanol acetate, acetic acid propyl ester, ethyl propanoate, acetic acid ethyl ester-D, acetic acid ethyl ester-M, ethyl formate, ethyl (E)-2-butenoate, ethyl 2-methylpentanoate-D, and ethyl 2-methylpentanoate-M. The esters present in all samples enhance the fruity character of Musalais wine. In particular, esters and aldehydes with lower molecular weights are important components that contribute to the flavor profile of the wine. These compounds are primarily formed during fermentation as secondary aroma products [37]. Contact between esters in wine increases their total concentration, which is in agreement with the results of Sanchez-Palomo et al. (2006) [38] from wines with different brewing processes.

Ketone compounds are produced by the oxidation of unsaturated fatty acids, typically endowed to Musalais wine with floral and fruity aromas, and the floral aroma tends to intensify with the elongation of the C chain. Most ketone substances have high thresholds and low concentrations, contributing little to the flavor of mushrooms. However, their stable properties and enduring fragrance help enhance the overall flavor [39]. The ketone compounds with the highest contents are 3-octanone, 3-mercapto-2-butanone, 4-methyl-2-pentanone, and levo-carvone, which have fruity and fresh aromas, of which 4-methyl-2-pentanone is an important pharmaceutical intermediate. 3-octanone has a strong fruity odor and is a compound with a relatively low odor threshold compared to other ketones, which makes it a key VOC in the formation of the flavor of *Boletus edulis* [11,40]. However, He et al. (2022) [41] outlined VOC dynamics and their metabolic pathways in *Aspergillus niger*-infected paddies using GC-IMS and developed fungal prediction models based on the VOC analysis. In this study, 40 species of VOCs in uninfected paddies were detected by GC-IMS. Most of the VOCs declined after infection, but conversely, 3-octanone and 1-octen-3-ol increased.

As presented in Figure 2E, significant differences in VOCs were observed in the fingerprints of the Musalais wine samples, especially between the samples that fermented at 28 °C, 25 °C with 22 °C; in addition, their corresponding signal intensities were different. Butylbenzene, acetic acid ethyl ester-D, dimethyl disulfide, butanoic acid ethyl ester-D, and benzaldehyde have the highest contents in all nine samples, where ethyl acetate had the best fruity flavor; 2-furanmethanol acetate, acetic acid propyl ester, and ethyl propanoate imparted fruity or green flavor; and 1-heptanol, 4-methyl-2-pentanone, and 3-pentanone had relatively higher amounts, especially in the 28 °C-C samples in relation to the 28 °C-A, B; 25 °C-A, B, C; and 22 °C-A, B, C samples.

### 3.4. Multivariate Statistical Analysis of Volatile Substances

Based on the differences in VOCs, the peak volumes of 50 characteristic regions in the aforementioned fingerprint chromatograms were used as characterization variables to perform PCA on the nine Musalais wine samples. The contribution rate of the first principal component PC1 was 14.3% and that of the second principal component PC2 was 48.9%, with a cumulative contribution rate of 63.2% for the first two principal components (Figure 3A). This indicates that PC1 and PC2 collectively account for most of the information from the original variables and can represent the main characteristics of volatile flavors in different treatment groups [42]. The nine Musalais wine samples appear to be well separated according to the fermentation condition and brewing process (Figure 3A). As can be seen in Figure 3A, the distances between the 28 °C-B, C and 22 °C-A, B, C/ 25 °C-C samples are wide, indicating that the aroma characteristics of Musalais wines with different fermentation conditions were quite different from each other. This is consistent with the results of GC-IMS fingerprint spectrum analysis.

As shown in Figure 3A,D, the 22 °C-B, C sample exhibited high scores on positive PC1 and positive PC2, which contained high loadings of 4-methyl-2-pentanone, 3-pentanone, ethyl 2-methylpentanoate-D, 2-butanol, 2-furanmethanol,5-methyl, etc., indicating that these volatile compounds were more abundant in Musalais wine samples. The 22 °C-B exhibited high scores on positive PC1 and negative PC2, 2-methyl propyl acetate, acetic acid propyl ester, acetic acid ethyl ester-D, ethyl propanoate, ethyl 2-methylpentanoate-M, and 3-octanone. The 25 °C-A, B, C and 28 °C-A samples exhibited high scores in negative PC 1 and positive PC 2, 1,4-dioxan, (E)-3-hexenoic acid, ethyl 3- methylbutanoate-M, and 2-furanmethanol. In addition, 28 °C-B, C also demonstrated high scores in negative PC 1 and negative PC 2, with aroma compounds such as 1,3-dioxolane, 2,4-dimethylcis, 2-butoxyethanol, acetic acid ethyl ester-M, butanoic acid ethyl ester-M, ethyl 3-methylbutanoate-D, 3-nonen-1-ol, etc., among which linalool oxide II was the most abundant compound.

The OPLS-DA statistical method was used to further screen characteristic markers and distinguish the contribution of different VOCs to Musalais wine samples. Variable data and classification information were divided into two datasets, and differences between groups were mined with the help of grouping information through projection and discriminant calculations [43]. In Figure 3B, the PLS-DA score plot was drawn using the 50 identified VOCs in the four groups of samples as dependent variables and different treatment groups as independent variables. The plot showed the same trend as the PCA, with more concentrated samples between groups. By randomly changing the order of classification variables to establish corresponding models, R^2^X and R^2^Y represent the percentages of information in the X and Y matrices explained by the PLS-DA classification model, respectively. The closer their values are to 1, the better the model performance, and the difference between them should not be too large [40]. Q^2^, calculated through cross-validation, evaluates the predictive ability of the PLS-DA model, where a larger Q^2^ indicates better predictive performance. Figure 3C shows the performance index results of the model obtained through 200 sequential permutation tests in PLS-DA: the independent variable fitting index R^2^X is 0.977, the dependent variable fitting index R^2^Y is 0.966, |R^2^X-R^2^Y| is <0.15, and the model prediction index Q^2^ is 0.848. Additionally, the intersection point of the Q^2^ regression line with the vertical axis is less than 0, indicating no overfitting in the model and validating its effectiveness for the aroma discrimination analysis of the Musalais wine samples.

In the PLS-DA model, the variable importance for the projection (VIP) value was used to further distinguish the contribution of different aroma substances to Musalais wine of different fermentation treatments [44]. The horizontal axis of Figure 3D corresponds to the numbered VOCs in Table 1, and the vertical axis represents the VIP value of each substance. According to the criterion of VIP > 1, 19 characteristic marker substances were screened out, including 4 esters, 1 ketone, 6 alcohols, 1 alkene, 3 acids, 1 amine, 1 aromatic hydrocarbon, and 2 others. These substances are as follows. (1) Esters: 2-methyl propyl acetate, ethyl (E)-2-butenoate, ethyl 2-methyl pentanoate-D, and ethyl 2-methylpentanoate-M. (2) Ketones: 3-octanone. (3) Alcohols: 2-methylpropanol-M, 2-methylpropanol-D, 2-furanmethanol,5-methyl, 1-propanethiol, 3-nonen-1-ol, and 3-methylbutan-1-ol-M. (4) Alkene: 1,4-dioxan. (5) Acids: 2-methylheptanoic acid, (E)-3-hexenoic acid and 2-methylpropionic acid. (6) Amines: N-nitrosomethylethylamine. (7) Aromatic hydrocarbons: butylbenzene. (8) Others: tetrahydrofuran and pyrrolidine. These key compounds significantly influence VOC differentiation among Musalais wines and provide a scientific basis for distinguishing their flavor profiles under varying fermentation temperatures.

### 3.5. Cluster Analysis of VOCs from Musalais Wine with Different Brewing and Fermentation Conditions

**Figure 4:**
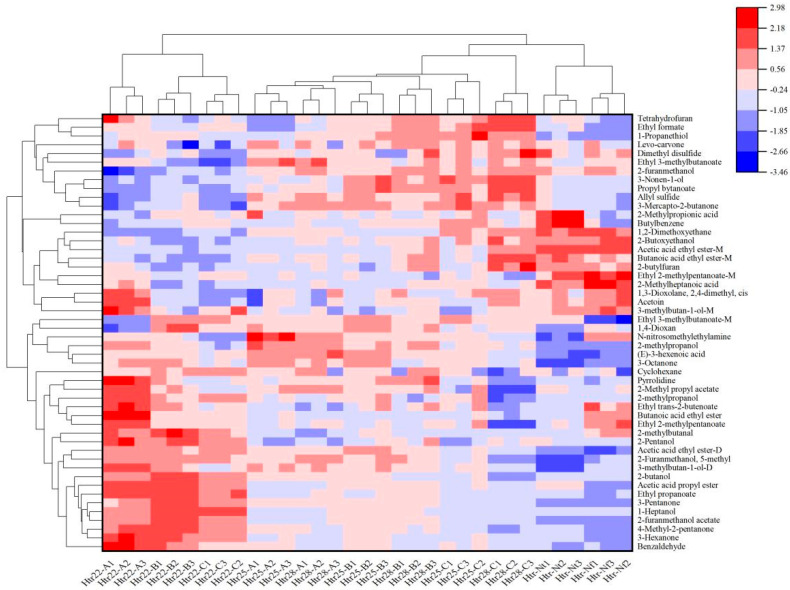
Heat map analysis of aromatic compounds with OAV > 1 in Musalais wines with different fermentation conditions.

Cluster analysis was performed on the VOCs of Musalais wine with different brewing and fermentation conditions. The results are shown in Figure 4, where the Y-axis represents the names of VOCs identified by GC-IMS, and the X-axis represents the origin codes. The VOCs of nine Musalais wine samples are clustered into two major groups: 22 °C-A, B and C belong to one group, while 25 °C-A, B, C and 28 °C-A, B, C belong to another. Of these, 22 °C-B and C are similar in flavor and form a group. The results of the cluster analysis are consistent with those of PCA, indicating that fermentation temperature and juice Brix may affect the formation of VOCs in Musalais wine.

### 3.6. Correlation Analysis Between Physicochemical Indices and Volatile Substances

**Figure 5:**
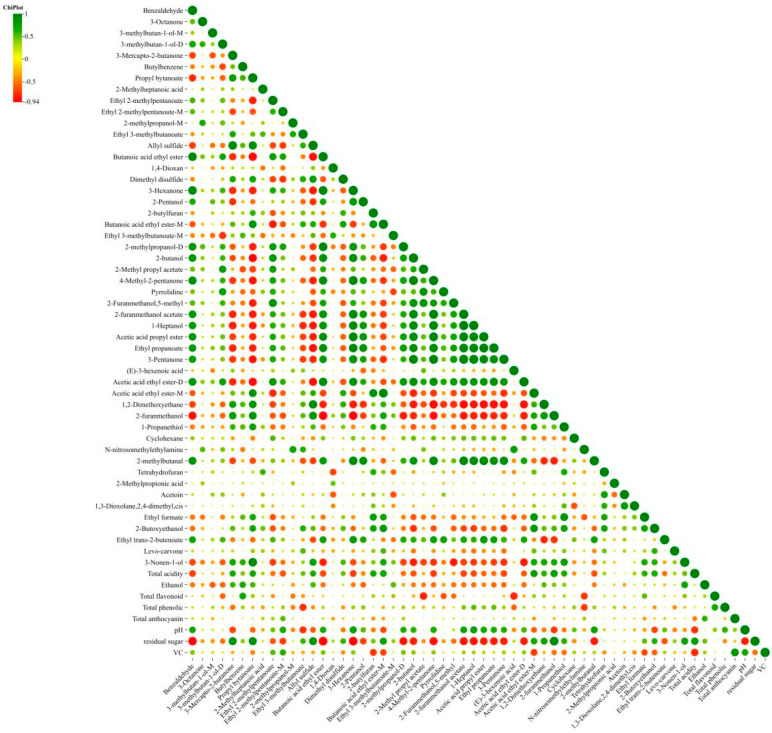
Correlation analysis between physicochemical indices and volatile substances.

Figure 5 illustrates correlations between VOCs and Musalais wine properties, where green denotes positive correlations, red indicates negative correlations, and larger points represent stronger associations [45]. Ethanol showed positive correlations with fruity/sulfurous compounds (3-mercapto-2-butanone, ethyl 3-methylbutanoate) but negative correlations with aldehydes and higher alcohols. Total acidity was positively associated with sulfur-containing compounds and esters; residual sugar was strongly correlated with similar compounds as total acidity; and pH and phenolic compounds showed opposite correlation patterns to acidity. Higher fermentation temperatures increased total acidity and residual sugar, whereas the lower temperatures (22 °C) enhanced phenolic and anthocyanin content and complex VOC profiles with fruity esters. Optimal balance achieved at 25 °C with 29 °Brix for ethanol production, antioxidant retention and moderate acidity. This demonstrated the temperature-dependent formation of flavor compounds, with 22 °C favoring aromatic complexity and 25 °C providing optimal balance between ethanol and phytochemical contents.

## 4. Conclusions

This study investigated the effects of fermentation temperature (22 °C, 25 °C, and 28 °C) and juice Brix (26 °, 29 °, 32 °) on Musalais wine quality. Higher temperatures increased total acidity (peaking at 8.24 ± 0.20 g/L at 28 °C/26 °Brix) and residual sugar, while pH reached maximum levels at 22 °C/32 °Brix. Optimal ethanol production occurred at 25 °C. Antioxidant components varied with temperature: VC and anthocyanins peaked at 25 °C, while total phenolics were highest at 22 °C/32 °Brix. Flavonoid content depended primarily on Brix level, maximizing at 32 °Brix. GC-IMS analysis identified 50 volatile compounds, with esters (30%) being most abundant. The 22 °C fermentation produced the most complex VOC profile, characterized by butanoic acid ethyl ester and 4-methyl-2-pentanone. PCA distinguished wines by fermentation conditions, with 22 °C wines containing higher levels of ketones and 28 °C wines showing increased esters. PLS-DA identified 19 marker compounds, and hierarchical clustering revealed two distinct wine groups based on aroma profiles. Fermentation temperature influenced VOCs significantly more than Brix level. The 22 °C condition produced wines with the most complex aromas. These findings demonstrate the critical role of temperature control in optimizing Musalais wine quality.

## Figures and Tables

**Table 1:** Volatile compounds in Musalais wine from different brewing conditions.

Serial Number	Volatile Compounds	Aroma Description	CAS Number	Molecular Formula	RI	RT/s	Dt/ms
Aldehydes							
1	Benzaldehyde	Almond-like, sweet, caramel-like	C100527	C_7_H_6_O	1478.2	931.37	1.49817
2	2-methylbutanal	Pungent, malty–nutty	C96173	C_5_H_10_O	844.1	161.886	1.15319
Alcohols							
3	2-methylpropanol-M	Jasmine aroma, apple aroma, rose aroma	C78831	C_4_H_10_O	1106.3	330.378	1.37197
4	2-Pentanol	A pungent, fruity/alcoholic	C6032297	C_5_H_12_O	1092.2	315.058	1.205
5	2-methylpropanol-D	subtle apple aroma	C78831	C_4_H_10_O	1052.2	277.953	1.38121
6	2-butanol	Almond-like, sweet, caramel-like	C78922	C_4_H_10_O	1034.8	263.244	1.34048
7	2-Furanmethanol, 5-methyl	Caramel, bread-like aroma	C3857258	C_6_H_8_O_2_	982.7	225.841	1.2658
8	1-Heptanol	Heavy, oily, green	C111706	C_7_H_16_O	976.3	222.389	1.39717
9	2-furanmethanol	Caramel, bread-like aroma	C98000	C_5_H_6_O_2_	882.1	177.387	1.12604
10	1-Propanethiol	Intensely pungent, sulfurous aroma	C107039	C_3_H_8_S	844.1	161.907	1.17181
11	3-Nonen-1-ol	Cucumber–melon freshness	C10340235	C_9_H_18_O	1158.7	396.965	1.37738
12	3-methylbutan-1-ol-D	pungent, fruity/alcoholic	C123513	C_5_H_12_O	1221.7	492.456	1.49817
13	3-methylbutan-1-ol-M	A pungent, fruity–fusel aroma with complex alcoholic and fermented nuances	C123513	C_5_H_12_O	1231.1	508.095	1.24796
Ketones							
14	3-Octanone	Creaminess, rose, jasmine	C106683	C_8_H1_6_O	1241.1	525.188	1.31526
15	3-Mercapto-2-butanone	Pungent, savory, sulfurous	C40789988	C_4_H_8_OS	1267.4	573.086	1.12763
16	3-Hexanone	Green, fruity, slightly pungent	C589388	C_6_H_12_O	1052.4	278.16	1.45111
17	4-Methyl-2-pentanone	Sharp, sweet, solvent-like	C108101	C_6_H_12_O	1033.2	261.984	1.47626
18	3-Pentanone	Mild, sweet, ethereal	C96220	C_5_H_10_O	998.9	235.309	1.35641
19	Levo-carvone	Cool, herbaceous, subtly sweet	C6485401	C_10_H_14_O	1213.1	478.463	1.31052
Esters							
20	Propyl butanoate	Banana scent, pineapple scent	C105668	C_7_H_14_O_2_	1154.6	391.363	1.25786
21	Ethyl 3-methylbutanoate-D	Refined, ultra-fruity	C108645	C_7_H_14_O_2_	1116.9	342.873	1.24933
22	Butanoic acid ethyl ester-D	Apple aroma, buttery aroma	C105544	C_6_H_12_O_2_	1056.4	281.632	1.55951
23	Butanoic acid ethyl ester-M	Pineapple aroma, banana aroma	C105544	C_6_H_12_O_2_	1063.6	288.04	1.20243
24	Ethyl 3- methylbutanoate-M	Vibrantly fruity, tropical–sweet	C108645	C_7_H_14_O_2_	1054.1	279.634	1.27258
25	2-Methyl propyl acetate	Bright, fruity, slightly floral	C110190	C_6_H_12_O_2_	1029.6	259.042	1.61204
26	2-furanmethanol acetate	Warm, balsamic–sweet	C623176	C_7_H_8_O_3_	999.8	235.928	1.41516
27	Acetic acid propyl ester	Fresh, fruity, slightly herbal	C109604	C_5_H_10_O_2_	993.8	231.938	1.48052
28	Ethyl propanoate	Bright, fruity, and rum-like	C105373	C_5_H_10_O_2_	974.7	221.546	1.45274
29	Acetic acid ethyl ester-D	Fruity-sweet, wine-like, brandy undertone	C141786	C_4_H_8_O_2_	902.9	186.439	1.34715
30	Acetic acid ethyl ester-M	Fruity, pineapple-like, sweet, green, waxy	C141786	C_4_H_8_O_2_	892.1	181.664	1.09893
31	Ethyl formate	Sharp, fruity–ethereal	C109944	C_3_H_6_O_2_	849.8	164.123	1.22488
32	ethyl (E)-2-butenoate	Sharp, fruity–pungent	C623701	C_6_H_10_O_2_	1141.7	373.98	1.54672
33	Ethyl 2-methylpentanoate-D	Bright, fruity, slightly herbal	C39255328	C_8_H_16_O_2_	1139.7	371.392	1.74581
34	Ethyl 2-methylpentanoate-M	Fruity, tropical, slightly green	C39255328	C_8_H_16_O_2_	1141.1	373.325	1.30848
Acids							
35	2-Methylheptanoic acid	Pungent, earthy–musky, faintly fruity	C1188029	C_8_H_16_O_2_	1145.1	378.479	1.40971
36	(E)-3-hexenoic acid	Green, fatty, slightly sweaty	C1577180	C_6_H_10_O_2_	1000.4	236.432	1.2286
37	2-Methylpropionic acid	Vinegary with a buttery undertone, sharp, sweaty, dairy-like	C79312	C_4_H_8_O_2_	784.8	140.397	1.15387
Ethers							
38	Allyl sulfide	Pungent, intensely garlic-like	C592881	C_6_H_10_S	1121.1	348.048	1.13028
39	Dimethyl disulfide	Pungent, sulfurous aroma	C624920	C_2_H_6_S_2_	1092.1	315	1.13104
40	1,2-Dimethoxyethane	Sweetly ethereal, faintly fruity	C110714	C_4_H_10_O_2_	930.8	199.358	1.09708
41	2-Butoxyethanol	Mild, sweet, slightly floral–ethereal	C111762	C_6_H_14_O_2_	893.1	182.097	1.20459
Alkanes							
42	Cyclohexane	Mild, sweet	C110827	C_6_H_12_	709.5	117.184	1.11184
43	1,4-Dioxan	Etheric/clean, slightly sweet and fruity	C123911	C_4_H_8_O_2_	1097.3	320.214	1.13104
Amines							
44	N-nitrosomethylethylamine	Slightly sweet but offensive	C10595956	C_3_H_8_N_2_O	841.6	160.951	1.11657
Aromatic hydrocarbons							
45	Butylbenzene	Neroli, jasmine, pineapple	C104518	C_10_H_14_	1301.3	636.777	1.20606
others							
46	Tetrahydrofuran	Ethereal, sweet, and slightly pungent	C109999	C_4_H_8_O	851.2	164.698	1.06385
47	Acetoin	Buttery or creamy, caramel, vanilla, sweet	C513860	C_4_H_8_O_2_	731.1	123.429	1.34026
48	1,3-Dioxolane, 2,4-dimethyl, cis	Sweet and fruity, scent of fresh flowers	C3390123	C_5_H10O_2_	734.5	124.436	1.3821
49	2-butylfuran	Overripe pear or dried fruit	C4466244	C_8_H_12_O	1110.4	335.237	1.17524
50	Pyrrolidine	Strong, sharp odor	C123751	C_4_H_9_N	998.6	235.087	1.27258

## Data Availability

The original contributions presented in the study are included in the article, further inquiries can be directed to the corresponding author.

## References

[1] Izquierdo-Cañas P.M., González Viñas M.A., Mena-Morales A., Pérez Navarro J., García-Romero E., Marchante-Cuevas L., Gómez-Alonso S., Sánchez-Palomo E. (2020). Effect of fermentation temperature on volatile compounds of Petit Verdot red wines from the Spanish region of La Mancha (central-southeastern Spain). Eur. Food Res. Technol..

[2] Spranger M.I., Climaco M.C., Sun B., Eiriz N., Fortunato C., Nunes A., Leandro C., Avelar M.L., Belchior A.P. (2004). Differentiation of red winemaking technologies by phenolic and volatile composition. Anal. Chim. Acta.

[3] Tang R., Abudureheman B., Zhang J.L., Chen L., Li H.Y., Zhu S., Guo M.Q., Huang J.L., Zhu X., Ye X.Q. (2025). Fermentation dynamics: Microbial and metabolite shifts in Musalais wine. J. Agric. Food Res..

[4] Yang F., Chen C., Ni D.R., Yang Y.B., Tian J.H., Li Y.Y., Chen S.G., Ye X.Q., Wang L. (2023). Effects of fermentation on bioactivity and the composition of polyphenols contained in polyphenol-rich foods: A Review. Foods.

[5] Du X., Myracle A.D. (2018). Fermentation alters the bioaccessible phenolic compounds and increases the alpha-glucosidase inhibitory effects of aronia juice in a dairy matrix following in vitro digestion. Food Funct..

[6] Zhu S.L., Chen Q.L., Zhang W.X.E., He J., Guo S.T., Kan L.P., Yang X.Y. (2025). Effect of different fermentation methods on the aroma and sensory characteristics of Musalais wine. Zhongguo Niangzao.

[7] Wu R.M., Chen Q.L., Aypari A., Guliziba A., Zhang X., Yang X.Y. (2024). Fermentation process optimization and quality analysis of red grape Musalais in Xinjiang. Zhongguo Niangzao.

[8] Li Z.J., Zhang X.D., Long B.X., Cao Y., Hou X.J. (2025). Research on product development and response surface optimization of Msalais pear. Agric. Prod. Process..

[9] Zhu L.X., Wang L.L., Song H.Z., Guo D.Q., Fan Y.G., Hou C.H., Xue J.L. (2012). Qualitative analysis of the main aroma compounds associated with traditional Musalais processing in Xinjiang, China. J. Inst. Brew..

[10] Gao X.Y., Bai Y.J., Zheng W.C., Chen L.H., Fan S.H., Feng Z.S. (2019). Study on the quality and aroma components of Musalais by different treatments. Food Mach..

[11] Zhang L., Zhang Y., Huang Y., Xuan Y., Hou X.J. (2018). Changes of volatile components in Musalais by E-nose and GC-MS. Sci. Technol. Food Ind..

[12] Yang B., He S., Liu Y., Liu B., Ju Y., Kang D., Sun X., Fang Y. (2020). Transcriptomics integrated with metabolomics reveals the effect of regulated deficit irrigation on anthocyanin biosynthesis in Cabernet Sauvignon grape berries. Food Chem..

[13] Cai Y.N., Li X., Ding J., Huang J.Y., Bao X.Y. (2021). Uncertainty evaluation for the determination of alcohol content in re-test compound wine by pycnometric method. J. Food Saf. Qual..

[14] Association of Official Analytical Chemists Inc. Changes in official methods of analysis of the Association of Official Analytical Chemists, Second supplement, 1991 to the fifteenth edition 1991, pp. 61–117. https://www.scirp.org/reference/referencespapers?referenceid=440931.

[15] Yadav D.K., Chand K., Kumari P. (2022). Effect of fermentation parameters on physicochemical and sensory properties of Burans wine. Syst. Microbiol. Biomanufacturing.

[16] Varo M.A., Martin-Gomez J., Serratosa M.P., Merida J. (2022). Effect of potassium metabisulphite and potassium bicarbonate on color, phenolic compounds, vitamin C and antioxidant activity of blueberry wine. LWT.

[17] Cheng Y., Wang Y., Yuan T., Xie J., Yu Q., Chen Y. (2024). Polyphenol compounds contributing to the improved bioactivities of fermented *Rubus chingii* Hu. Food Res. Int..

[18] Sharma A., Yadav M., Sharma N., Kumari A., Kaur S., Meenu M., Garg M. (2022). Comparison of wheatgrass juices from colored wheat (white, black, blue, and purple) for health promoting phytochemicals. Food Res. Int..

[19] Jiang P., Miao X., Li J., Qi H., Shang S., Dong X. (2024). Volatile flavor characteristics of scallops (*Chlamys farreri*) with different drying methods were analyzed based on GC-IMS and GC-O-QTOF. Food Chem. X.

[20] Liu W. (2005). A Study on Ohmic Heating Properties and Its Physical-Chemical Characters of Fruit Juice with Fruit Particles. Ph.D. Thesis.

[21] Du Q., Zhi R.J., Zang X.M., Qu R., Ye D.Q., Nan H., Liu Y.L. (2024). Reshaping yeast metabolism and enhancing the quality of fresh-style red wine through low-temperature fermentation. LWT-Food Sci. Technol..

[22] Don S.M., Rambli M., Nore B.F. (2024). Antioxidant content following fermentation of lemongrass for herbal beverage development. J. Food Sci. Technol..

[23] Zhang J., Chen D., Chen X., Kilmartin P., Quek S.Y. (2019). The influence of vinification methods and cultivars on the volatile and phenolic profiles of fermented alcoholic beverages from cranberry. Antioxidants.

[24] Ampofo J., Ngadi M., Ramaswamy H.S. (2020). The impact of temperature treatments on elicitation of the phenylpropanoid pathway, phenolic accumulations and antioxidative capacities of common bean (*Phaseolus vulgaris*) sprouts. Food Bioprocess Technol..

[25] Zhou Q.E., Wang X.Y., Tian C.R., Guo Y.R. (2011). Comparison of enological characteristics between fresh apple juice and juice concentrate. Sci. Technol. Food Ind..

[26] Zhang H., Jiang Y., Lv Y., Pan J., Duan Y., Huang Y., Geng K. (2017). Effect of water quality on the main components in Fuding white tea infusions. J. Food Sci. Technol..

[27] Qiao T.T., Zhu L.X. (2023). Aroma threshold and binary synergy of furanone, 5-methylfurfural and 3-methylthiopropanol in Musalais wine. Food Sci..

[28] Miao X., Li S., Shang S., Sun N., Dong X., Jiang P. (2023). Characterization of volatile flavor compounds from fish maw soaked in five different seasonings. Food Chem. X.

[29] Lu X.K., Yang B., Sun Y., He J.N., Fan B.M., Sun H., Sun X.T. (2023). Feature analysis and identification of Baijiu based on gas chromatography-ion migration spectrometry. J. Chin. Inst. Food Sci. Technol..

[30] Hou R., Jelley R.E., van Leeuwen K.A., Pinu F.R., Fedrizzi B., Deed R.C. (2023). Hydrogen sulfide production during early yeast fermentation correlates with volatile sulfur compound biogenesis but not thiol release. FEMS Yeast Res..

[31] Kopriva S., Malagoli M., Takahashi H. (2019). Sulfur nutrition: Impacts on plant development, metabolism, and stress responses. J. Exp. Bot..

[32] Sirisena S., Chan S., Roberts N., Dal Maso S., Gras S.L., Martin G. (2024). Influence of yeast growth conditions and proteolytic enzymes on the amino acid profiles of yeast hydrolysates: Implications for taste and nutrition. Food Chem..

[33] Xu Y., Wang D., Li G., Hao J., Jiang W., Liu Z., Qin Q. (2017). Flavor contribution of esters in lager beers and an analysis of their flavor thresholds. J. Am. Soc. Brew. Chem..

[34] Alves V., Gonçalves J., Figueira J.A., Ornelas L.P., Branco R.N., Câmara J.S., Pereira J.A. (2020). Beer volatile fingerprinting at different brewing steps. Food Chem..

[35] Wang F., Zhao P., Du G., Zhai J., Guo Y., Wang X. (2024). Advancements and challenges for brewing aroma-enhancement fruit wines: Microbial metabolizing and brewing techniques. Food Chem..

[36] Alises M.O., Sanchez-Palomo E., Gonz’alez-Vinas M.A. (2023). Effects of winemaking techniques on the volatile compounds of Chelva wines. Food Biosci..

[37] Cao W.Y., Shu N., Yang Y.M., Wen J.L., Lu W.P. (2023). Comprehensive evaluation of nine grape varieties based on fundamental physical and chemical indicators, color and volatile compounds. Res. Rep..

[38] Sanchez-Palomo E., Perez-Coello M.S., Díaz-Maroto M.C., Gonzalez-Vinas M.A., Cabezudo M.D. (2006). Contribution of free and glycosidically-bound volatile compounds to the aroma of muscat “a petit grains” wines and effect of skin contact. Food Chem..

[39] Xu N., Lu H., Feng L.G., Huang X.-H. (2020). Volatile components analysed by HS-SPME-GC-MS in different parts of fruiting bodies and nutritional composition of *Oudemansiella raphanipes*. Mycosystema.

[40] Jin W.G., Liu J.X., Sun H.Y., He L.L., Pei J.J., Cheng H., Jiang P.F. (2023). Characterization of volatile organic compounds of Giant Salamander (*Andrias davidianus*) oil adulterated with different amounts of peanut oil by gas chromatography-ion mobility spectrometry combined with chemometrics. Food Sci..

[41] He P., Hassan M.M., Tang F., Jiang H., Chen M., Liu R., Lin H., Chen Q. (2022). Total fungi counts and metabolic dynamics of volatile organic compounds in paddy contaminated by *Aspergillus niger* during storage employing gas chromatography-ion mobility spectrometry. Food Anal. Methods.

[42] Shao S.X., Xu M.M., Lin Y.P., Chen X.M., Fang D.Y., Cai J.Y., Wang J.H., Jin S., Ye N.X. (2023). Differential analysis of aroma components of Huangguanyin Oolong tea from different geographical origins using electronic nose and headspace solid-phase microextraction-gas chromatography-mass spectrometry. Food Sci..

[43] Ni R.J., Zhang P., Tian H.L. (2022). Effects of frying time on volatile flavor compounds in fried pepper (*Zanthoxylum bungeanum*) oil as analyzed by gas chromatography-ion mobility spectrometry and multivariate statistical analysis. Food Sci..

[44] Zhang Y., Tong X.Y., Chen B.J., Wu S.H., Wang X., Zheng Q., Jiang F., Qiao Y.J. (2023). Novel application of HS-GC-IMS for characteristic fingerprints and flavor compound variations in citrus reticulatae pericarpium during storage with different *Aspergillus niger* fermentation. Food Chem. X.

[45] Jiang C., Liu Y., Jin W., Zhu K., Miao X., Dong X., Jiang P. (2024). Effects of curing concentration and drying time on flavor and microorganisms in dry salted Spanish mackerel. Food Chem. X.

